# Biomimetic Chemocatalytic Cascades—A New Strategy for Molecular Design of Degradable Polymer Systems

**DOI:** 10.1021/acs.macromol.4c02241

**Published:** 2025-01-13

**Authors:** Bin Tan, John R. Dorgan

**Affiliations:** 1 Department of Chemical Engineering and Materials Science, Michigan State University, East Lansing, Michigan 48824, United States; 2 Department of Materials Science and Engineering, Fujian University of Technology, Fuzhou 350011, China

## Abstract

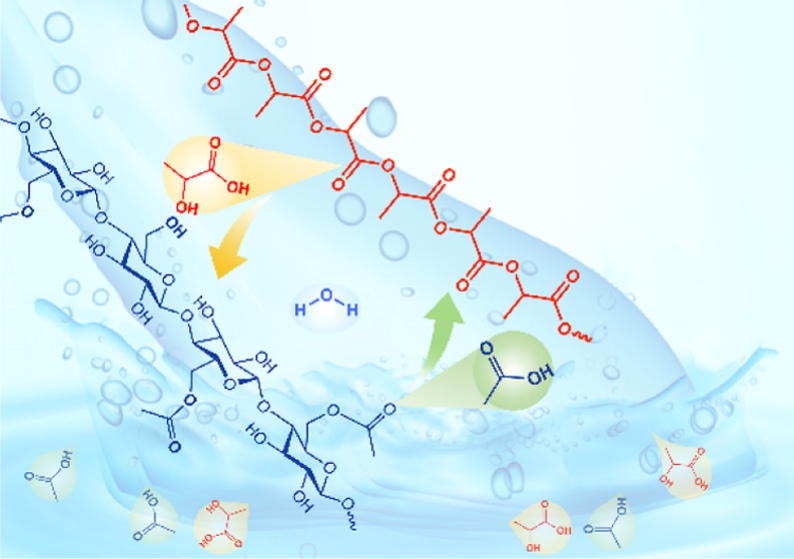

Biological systems often involve cascading molecular signals; for example, blood coagulation involves a cascade of serial and parallel reactions catalyzed by enzymes. The present study draws inspiration from such complex biological systems to demonstrate, through a simple example, the purposeful design of a cascade system that enables control over polymer degradation kinetics. Micron size fibers of polylactide (PLA), cellulose acetate (CA), and their mixtures are subjected to hydrolysis at varying temperatures. Cleavage of the PLA produces an organic acid functional group that catalyzes the CA hydrolysis, thus demonstrating the use of synthetic molecular signaling. Furthermore, the presence of CA inhibits the degradation of PLA thereby demonstrating molecular feedback, another hallmark of biological molecular cascades. The parallel reaction cascade causes the hydrolysis rate constant for CA to increase 3.1 times compared to CA alone (from 5.7 × 10^–4^ to 1.78 × 10^–3^ L^2^ mol^–2^ h^–1^ at 125 °C); furthermore, due to molecular feedback, the hydrolysis rate constant for PLA decreases by 21% (from 2.40 × 10^–3^ to 1.90 × 10^–3^ L^2^ mol^–2^ h^–1^). The results demonstrate that synthetic signaling enables exquisitely tunable degradation kinetics. Technological applications of such purposely designed biomimetic systems are wide ranging and include the design of polymer systems for hydraulic fracturing, for biomedical applications, and for facilitating the recycling of mixed plastic wastes.

## 1 Introduction

Living systems use enzyme catalysts to execute both sequential and parallel reactions;^1^ such biosynthetic pathways are referred to as enzyme *cascades*.^2,3^ Advances in biocatalysis are defining new developments in synthetic biology, and there is an increasing ability to manipulate cascading sequences of enzymatic transformations. Enzyme cascades in chemical synthesis to allow “one-pot” methods have been explored.^4,5^ Artificial enzyme cascades of impressive complexity have been demonstrated over the past decades.^6−8^

The present study shows that chemocatalytic cascades can be designed to enhance polymer degradation. A synthetic cascade is demonstrated by using two polymers that undergo hydrolysis; this approach enables a clear demonstration of both direct and feedback molecular signaling in a synthetically derived polymer system. To realize this goal, cellulose acetate (CA) and poly(lactic acid) (PLA) fibers are hydrolyzed at temperatures above 100 °C.

CA is derived from the biopolymer cellulose through acetylation. Hydrolysis of CA involves relatively fast deacetylation and subsequent depolymerization of the cellulose backbone via the cleavage of the β_1–4_ glycosidic bond.^9−11^ Higher temperatures and strong acid or base conditions can promote the hydrolysis rate of CA.^10,12−16^ For instance, Vos et al. conducted hydrolysis of CA over a range of temperatures (23–95 °C) and found that there is a “V”-shaped relationship between the hydrolysis rate and pH (i.e., catalysis can be either acid or base driven).^13^

Polylactide (PLA) is a biodegradable polymer obtained by polymerizing a lactide monomer. The lactide is a cyclic diester of lactic acid that typically comes from fermentation of renewable plant-based sugars. The degradability of PLA is a crucial property that enables important applications, including biomedical sutures, controlled release devices, compostable plastics packaging, and degradable oilfield products.^17−19^ PLA degradation under varied conditions has been widely investigated.^20,21^ Generally, PLA is degraded by the hydrolysis of backbone ester bonds leading to random chain scission and the formation of carboxylic acids, a process which is autocatalytic.^22−25^ PLA hydrolysis proceeds faster than CA hydrolysis under the same hydrolysis conditions.

PLA/CA mixtures were used to create a chemocascade-enabled control over the rate of degradation of both polymers. Lactic and acetic acids have different strengths (different dissociation constants, *K*_a_) that affect the pH value of an unbuffered reaction medium differently. Therefore, the influence of the acidic groups generated catalyzes the hydrolysis of the remaining ester bonds differently compared to the individual polymers. In the language of enzymes, acetic acid generated by the hydrolysis of CA provides a cofactor for the hydrolysis of PLA. For demonstration purposes, fine fibers of PLA and CA (diameters on the order of 10 μm) and their mixtures were hydrolyzed over a temperature range from 100 to 175 °C. PLA molecular weight changes and acetic and lactic acid production were measured and analyzed to determine the influence of synthetic molecular signaling on hydrolysis kinetics.

## 2 Experimental Section

### 2.1 Materials

CA with an average acetyl content of 39.4 wt % (DS = 2.48) was obtained from the Eastman Chemical Company in the form of chopped fibers having lengths of 6 mm and diameters of 22 ± 5 μm. PLA fibers were provided by the Greenstar Company in the form of 5 mm long chopped fibers with a diameter of around 15 ± 5 μm. The small fiber diameter ensures that the measured hydrolysis rates are in the reaction-limited regime so that the results are free of diffusional limitations (see the Supporting Information).

### 2.2 Hydrolysis

Hydrolysis was carried out in 30 mL sealed stainless-steel tubular reactors. For a specific specimen, 10 g of fibers (either 10 g of PLA fibers, 5 g of PLA with 5 g of CA fibers, or 10 g of CA fibers) and 10 g of DI water were loaded into the reactors. Samples were purged with argon to prevent oxidative degradation and then placed into a preheated oven resting upon a VWR 3500 orbital shaker and continuously agitated (at 75 rpm). Hydrolysis reactions were conducted at 100, 125, 150, and 175 °C. To study kinetics, the small tubular reactors were removed from the oven and quenched in an ice water bath at varying times. The aqueous solution was recovered, and its pH was determined; lactic acid and acetic acid concentrations were also measured.

### 2.3 Characterization

The molecular weight of PLA was measured using a gel permeation chromatography (GPC) system composed of a multiangle laser light scattering instrument (DAWN, Wyatt technology) and an interferometric refractometer (OPTILAB, Wyatt technology). Chloroform was used as the solvent. Dilute solutions were filtered through a 0.22 μm syringe filter before injection into the GPC system, and the eluent flow rate was fixed at 1 mL/min. The pH values of aqueous solution after hydrolysis were measured in triplicate at room temperature using a calibrated electronic pH meter (FiveEasy F20); average values were reported.

The concentrations of lactic and acetic acids after hydrolysis were measured by liquid chromatography–mass spectrometry (LC–MS, Waters Acquity LC system coupled to a Waters TQ-D triple quadrupole mass spectrometer) and gas chromatography-mass spectrometry (GC–MS, Agilent 6890 GC/5975B MS system), respectively. For the LC–MS measurement, the LC flow rate was 0.5 mL/min. Sample ionization was via negative-mode electrospray ionization (ESI), with source parameters including an ESI voltage of −3 kV, a source temperature of 150 °C, a desolvation temperature of 500 °C, a cone gas flow of 45 L/h, and a desolvation gas flow of 800 L/h. For GC–MS measurement, 1 μL of the sample was delivered into the GC inlet, which was maintained at 250 °C with a 10:1 split flow and a 1 min purge time. The temperature of the GC oven was gradually raised from 50 to 120 °C at 7 °C/min, followed by heating to 240 °C at 40 °C/min and held for 3 min to ensure a clean baseline.

## 3 Results and Discussion

### 3.1 pH Values

**Figure 1:**
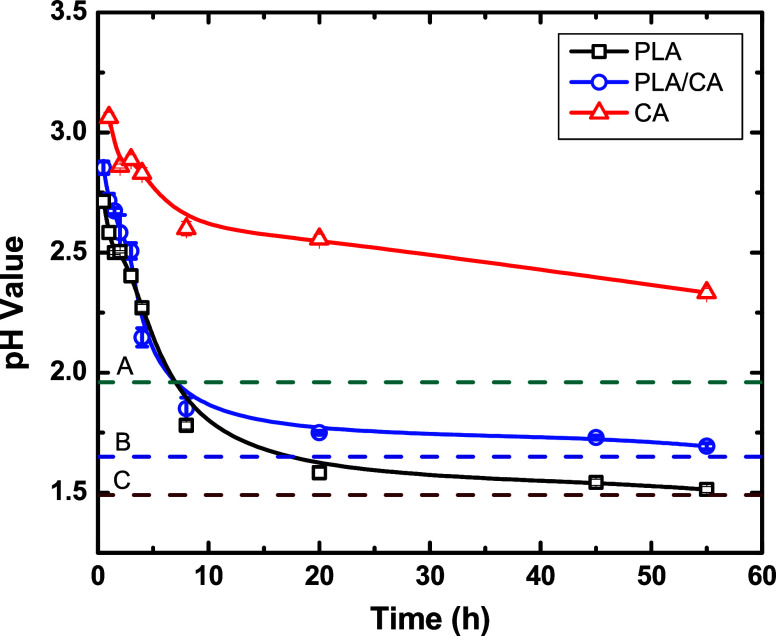
Hydrolysis enhancement via synthetic signaling in a purposely designed chemocatalytic cascade. Time variation of the pH during hydrolysis of PLA, CA, and a 50/50 PLA/CA mixture at 125 °C. The pH change of the mixture is not the simple weighted average of its individual components—degradation is enhanced through chemical cascading. At 55 h, CA retains many ester linkages, whereas in mixtures, the CA is fully deacetylated (dotted lines A, B, and C are the calculated pH values for CA, PLA/CA, and PLA assuming complete ester-bond hydrolysis).

Representative pH values of aqueous solutions during hydrolysis are shown in Figure 1. In addition to the organic acid functional group resulting from cleaving a PLA chain in the backbone, both acetic and lactic acids are generated by hydrolysis. The resulting increase in acidity causes pH values to decrease with increasing reaction time. Figure 1 shows that at 125 °C, the PLA/CA mixture produces a faster drop and lower pH when compared against CA. The hydrolysis of PLA enhances the acidity of the reaction environment, which serves as a signaling message to accelerate the hydrolysis rate of CA. Higher temperatures accelerate hydrolysis leading to faster drops in pH.

### 3.2 Kinetics of Hydrolysis

PLA hydrolysis depends on conditions and the physical form of the polymer being degraded. Various parameters, including crystallinity, morphology, and hydrolysis conditions, have been reported as affecting the degradation rate.^20^ Typically, the temperature is reported as having the most significant influence. It should be understood that in many studies, the conversion rate does not correspond to a true rate of chemical reaction but is instead simply an “apparent rate” which includes effects such as mass transfer (i.e., diffusional limitations of either reactants or products). In the present study, care has been taken to use fine fibers with small diameters so the experiments are conducted in the reaction rate-limited regime where true chemical kinetics can be measured (see the Supporting Information).

**Figure 2:**
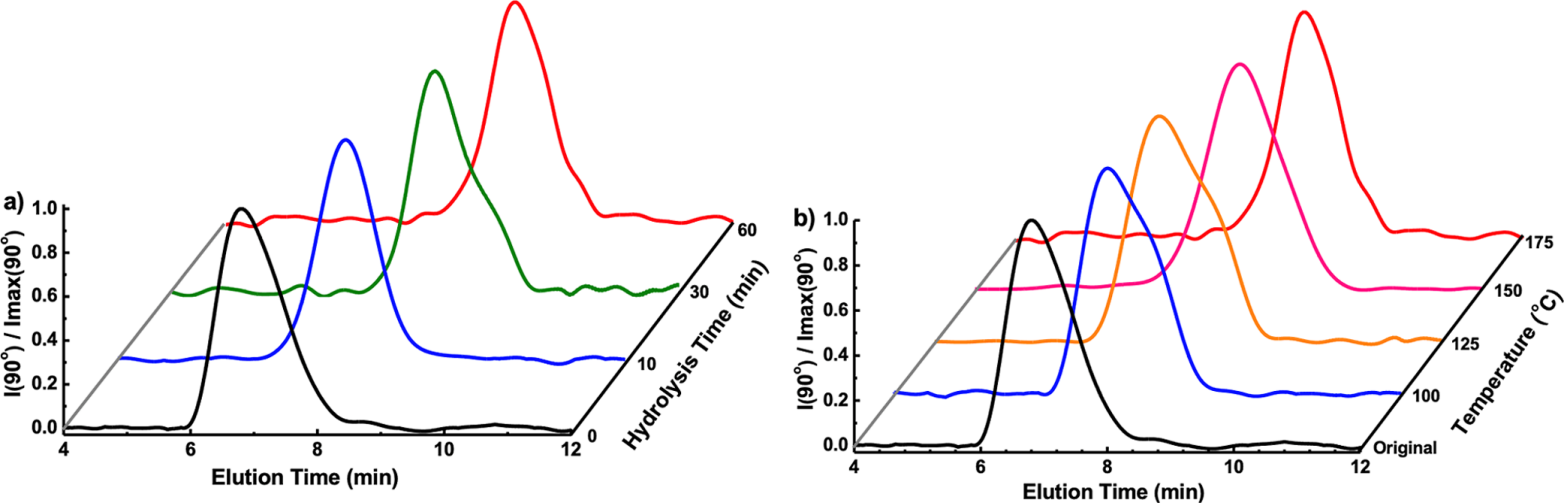
Molecular weight time evolution during PLA hydrolysis. Light scattering curves for PLA in PLA/CA mixtures hydrolyzed (a) at 175 °C for varying times and (b) for 1 h at varying temperatures.

Early in the hydrolysis process, degradation is dominated by chain scission of PLA. Only small amounts of free acids are generated, and the mass of recovered fibers is constant within the precision of the measurements. However, the molecular weight of recovered PLA decreases. This period during which the recoverable mass is constant persists for up to 8 h at 100 °C, and it decreases to about 4 h at 125 °C, 2 h at 150 °C, and only 90 min at 175 °C. GPC trace curves for PLA are presented in Figure 2; peaks shift to longer elution times, corresponding to lower molecular weights as either reaction time or temperature increases.

Ester hydrolysis can be described as a third-order reaction, where the reaction rate is dependent on the concentrations of water, ester bonds, and acid groups. Hydrolysis of polyesters is therefore an *autocatalytic* reaction—the acid product of reaction acts as a catalyst to further promote the hydrolysis process. Following Flory’s original treatment based on the equal reactivity of ester bonds, a detailed treatment of the chemical kinetics is presented in the Supporting Information.^26^ Briefly, the rate may be expressed by eq 1
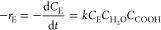
1Here, –*r*_E_ is the rate at which ester bonds are consumed and  are the molar concentrations of ester bonds, water, and acid, respectively. The symbol *k* is the rate constant for the hydrolysis of ester bonds. Hydrolysis of polyesters at early reaction times is often reported in terms of an apparent rate constant (; this is done to relate the reaction rate to changes in the molecular weight as written in the form of eq 2
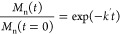
2where *M*_n_ is the number-average molecular weight and *k*^′^ is the apparent rate constant.

**Figure 3:**
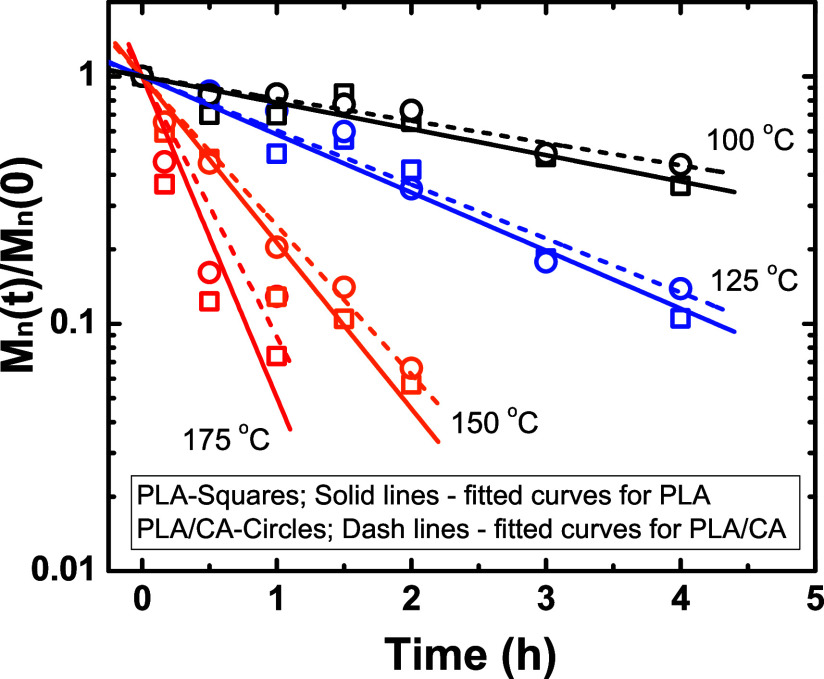
*M*_n_(*t*)/*M*_n_(0) versus time for PLA hydrolyzed at different temperatures. Solid and dashed lines are fit to data for PLA and PLA/CA mixtures, respectively.

Plots of *M*_n_(*t*)/*M*_n_(0) versus *t* are presented in Figure 3 for different reaction temperatures; the corresponding apparent rate constants are listed in Table 1. The differences in apparent rate constants (*k*^′^) between PLA and PLA/CA mixtures in this initial stage are insignificant; the values agree within the experimental uncertainties. Such a finding is consistent with the known autocatalytic nature of the hydrolysis. As demonstrated in Figure 1, in the initial stages (first few hours), there is little acid generated so minimal catalysis occurs. That is, there is an *incubation period*. As expected, temperature has a strong effect, and the rate constant increases 20 times as temperature increases from 100 to 175 °C.

**Table 1:** Apparent Rate Constants Determined by Molecular Weight Measurements at Early Times

	temperature (°C)	100	125	150	175
PLA	*k*^′^ (h^–1^)	0.24 ± 0.03	0.51 ± 0.05	1.89 ± 0.20	5.52 ± 0.55
PLA/CA	*k*^′^ (h^–1^)	0.20 ± 0.02	0.44 ± 0.05	1.65 ± 0.17	4.17 ± 0.42

Almost all literature studies on PLA report the apparent hydrolysis rate constant based on eq 2. Because of the dependence of the apparent rate on the initial stoichiometry, this has led to confusion in the literature. For example, at physiological mimicking conditions (pH = 7.4, *T* = 37 °C), the reported apparent rate constants differ by orders of magnitude, from a low value of 2.5 × 10^–5^ h^–1^ to a high value of 4.0 × 10^–3^ h^–1^.^27,28^ While the apparent rate representation is a useful empiricism for fixed conditions, such an approach is insufficient for understanding the underlying fundamental chemical kinetics in a cascading system.

### 3.3 Parallel Cascade Reaction

**Figure 4:**
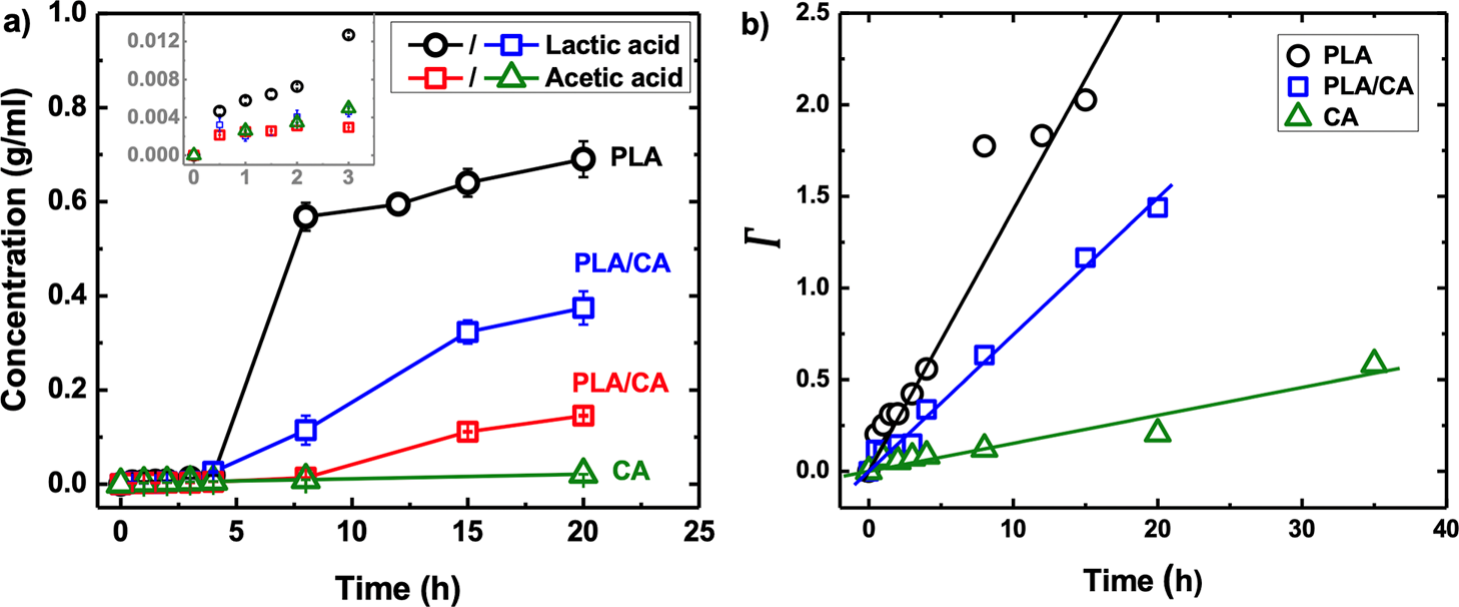
Kinetics of cascaded degradation at 125 °C. (a) Time evolution of lactic and acetic acid generation; when cascaded, the release rate of acetic acid increases and that of lactic acid decreases. (b) Analysis based on eq 5 of the Supporting Information to determine rate constants, and Γ represents the left side of eq S5.

Additional insight into the chemocascade kinetics is provided by examining the time evolution of the generated products. Lactic and acetic acid generated during hydrolysis at 125 °C were measured by LC-MASS and GC-MASS and results are shown in Figure 4. Combining the generated acid concentrations with the PLA molecular weight data provides a means of calculating the fractional conversion of all ester bonds as a function of time. Consistent with the pH and molecular weight measurements, Figure 4a shows an incubation stage of about 5 h during which minimal amounts of free acids are produced. Subsequently, the acetic acid concentration in PLA/CA mixtures is significantly higher than that for CA alone. That is, the presence of PLA and its release of the “co-factor” lactic acid leads to an accelerated production of acetic acid. Figure 4b presents plots based on eq 5 of the Supporting Information that allows determination of the hydrolysis rate constant (*k*) defined by eq 1.

Table 2 lists rate constants for PLA, PLA/CA mixtures, and CA at 125 °C. PLA proceeds the fastest (*k* = 2.40 × 10^–3^ L^2^ mol^–2^ h^–1^), followed by PLA/CA mixtures (*k* = 1.78 × 10^–3^ L^2^ mol^–2^ h^–1^), and CA is far slower (*k* = 5.7 × 10^–3^ L^2^ mol^–2^ h^–1^). In the PLA/CA mixtures, the rate of ester cleavage is 3.1 times greater than that in CA alone. Furthermore, as clearly evidenced in Figure 4a, the effect is not just a linear addition of the individual polymer degradation rates. At 8 h, the PLA sample produces a lactic acid concentration of 0.6 g/mL, whereas the mixture is only 0.1 g/mL (linear superposition would produce 0.3 g/mL). Similarly, at 15 h, the mixture produces an acetic acid concentration of 0.1 (g/mL), whereas CA alone produces an almost undetectable amount of acetic acid. Accordingly, the data unambiguously demonstrate that the chemocascade accelerates the rate of CA hydrolysis while moderating the rate of PLA hydrolysis. That is, the system exhibits the hallmarks of molecular signaling. To further investigate this phenomenon, the temperature dependence is explored.

**Table 2:** Rate Constants at 125 °C Calculated from Molecular Weight and Acid Concentration Data

fiber type	PLA	PLA/CA	CA
hydrolysis rate constant (*k*, × 10^–3^ L^2^ mol^–2^ h^–1^)	2.40 ± 0.12	1.78 ± 0.09	0.57 ± 0.03
*R*^2^	0.97	0.99	0.95

**Figure 5:**
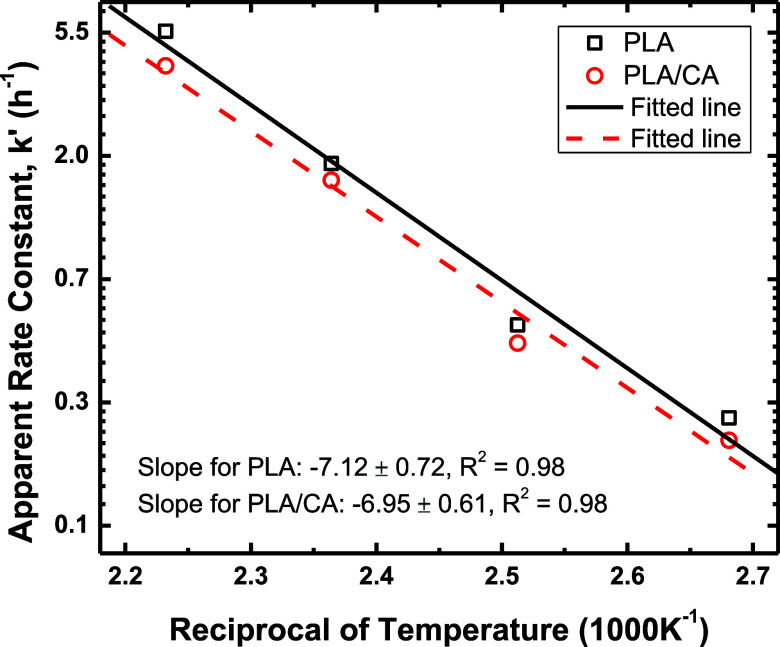
Arrhenius plot showing no distinction in observed activation energies for cascaded vs noncascaded systems.

The usual representation of temperature dependence of chemical kinetics rate parameters is given by eq 3, the Arrhenius equation
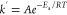
3Here, *k*^′^ is again the apparent rate constant, *E*_a_ is the activation energy, *R* is the universal gas constant, and *T* is the temperature. Because *k* and *k*^′^ are related by a fixed constant in the present set of experiments, either can be used; *k*^′^ is used to allow comparison with literature sources. An Arrhenius plot is presented in Figure 5; the data allow determination of activation energies to within about 10% and the determined value for PLA is 14.1 kcal/mol and that for the PLA/CA cascade is 13.8 kcal/mol. Accordingly, within the experimental uncertainties, there is no identifiable difference in the temperature dependence between the two cases and an appropriate value is *E*_a_ = 14 ± 1.4 kcal/mol.

The activation energy determined in the present case can be compared to the literature values. Reported values include 11.8 kcal/mol in the temperature range of 170–250 °C,^29^ 16.6 kcal/mol in the temperature range of 120–160 °C,^29^ and 20.8 kcal/mol in the temperature range of 100–130 °C.^30^ Given the experimental uncertainties, the first and second of these values seem reasonable, whereas the third one is out of the range (does not agree with the other 3 studies with in a 10% uncertainty) and should therefore be considered unreliable (note that the temperature range used is very small for that case).

As noted above, the use of an apparent rate constant can be problematic. Because its definition includes initial concentrations, comparison of values between different studies becomes difficult. If insufficient information is provided, then comparison becomes impossible. To make this point abundantly clear, reported literature values have been collected.^25,27−45^ At 37 °C, reported apparent rate constants for PLA hydrolysis vary by more than 2 orders of magnitude, from 2.5× 10^–5^ h^–1^ to 4.0 × 10^–3^ h^–1^. It is anticipated that in some of these reports, diffusional limitations were also important.

The collected and assessed rate constants from the literature enable further analysis of the temperature dependence on the rate of PLA hydrolysis. Data from 37 to 250 °C are presented in the Supporting Information (Figure S2). An Arrhenius analysis provides an activation energy of *E*_a_ = 16.7 ± 1.6 kcal/mol which agrees with the value of *E*_a_ = 14 ± 1.4 kcal/mol found in the present study. Given the lack of information about changing conditions and stoichiometries in the literature studies, it is suggested that the present value of *E*_a_ = 14 ± 1.4 kcal/mol be adopted.

The mechanism for the observed chemocatalytic effects is more complex than might be expected. Considering eq 1, if all acid and ester groups behaved the same, then there would be no difference in the observed rate constant. Certainly, a major effect is the difference in acidity of the generated products. Lactic acid has an acid dissociation constant of 1.38 × 10^–4^, while acetic acid has an acid dissociation constant of 1.58 × 10^–5^. Accordingly, lactic acid provides a stronger acidic environment that serves to catalyze CA hydrolysis to a greater extent than would otherwise occur; this is the primary method of molecular signaling. The CA is shown to hydrolyze faster. Correspondingly, when an ester bond from CA is hydrolyzed, the catalytic effect on PLA is greatly diminished compared to that if an ester from PLA had been broken; this serves as a form of molecular feedback that moderates the rate of PLA hydrolysis. However, there are a few nuances that can be detected in the data. For example, given that lactic acid is essentially 10× stronger, the effect of acetic acid on PLA could be considered negligible. This means that the PLA/CA experiments can be considered as being performed at “half strength”; that is, there is half as much PLA present in the cascade. Accordingly, one might expect, at least in the initial stages, that PLA would hydrolyze at half the rate. Instead, the process goes slower. The reason for this slow down can be found in eq 1. Namely, there are additional ester bonds available to be cleaved from CA. Thus, there are two distinct physical mechanisms responsible for the feedback leading to a slowed rate of PLA hydrolysis: one is simply the dilution effect, the less PLA present, the less acidic the environment. The second mechanism is a competition with CA for access to the catalyst and water; for a given rate of ester bonds being broken, a stoichiometric amount will be broken on both chains. By association with the acid catalysts and consumption of water, the CA signals its presence. Accordingly, the generated acids from the two separate reactions serve as cofactors in the parallel cascade. Additional investigation is underway to develop a kinetic model that explicitly considers the two ester cleaving reactions separately and to obtain separate rate constants based on the acid generation data.

## 4 Conclusions

The purposeful design of a chemocatalytic cascade that enables control of the polymer degradation rate is clearly demonstrated. PLA, CA, and their mixtures are subjected to hydrolysis at varying temperatures. Synthetic molecular signaling is achieved through cleavage of PLA to produce an organic acid functional group that catalyzes CA hydrolysis. Molecular feedback, a hallmark of biological cascades, is also present, the presence of CA inhibits the degradation rate of PLA. Compared to CA alone, the parallel reaction cascade causes the hydrolysis rate to increase by 3.1 times (from 5.7 × 10^–4^ to 1.78 × 10^–3^ L^2^ mol^–2^ h^–1^ at 125 °C).

The merit of such purposefully designed synthetic parallel cascades is the ability to select degradation rates. An exquisite level of molecular control is made possible. Technological applications of such purposely designed biomimetic systems are wide ranging and encompass degradable biological scaffolds, materials for hydraulic fracturing, and a foundational platform to design future plastics that facilitate the sustainable recycling of mixed plastic wastes.

## Supporting Information Available

Detailed derivation of kinetics model in terms of functional groups and fractional conversion; analysis of molecular weight during early stages of hydrolysis; comparison with other literature sources and confirmation of the activity energy for PLA hydrolysis; and verification of the particle size independence of the results indicating no mass transfer limitations (PDF)
