# Supplementary material for: Biomimetic Chemocatalytic Cascades—A New Strategy for Molecular Design of Degradable Polymer Systems
Source: Macromolecules. 2025 Jan 13;58(4):2046–52. doi: 10.1021/acs.macromol.4c02241 (PMC11866919; doi:10.1021/acs.macromol.4c02241)
Supplement: Supplementary file 1 — ma4c02241_si_001.pdf [file ma4c02241_si_001.pdf]

Supporting Information for

Biomimetic Chemocatalytic Cascades – A New Strategy for Molecular Design of Degradable Polymer Systems

Bin Tan and John R. Dorgan*
Department of Chemical Engineering and Materials Science, Michigan State University, East Lansing, MI 48824 USA

Kinetics for ester hydrolysis. Ester hydrolysis can be described as a catalyzed third order reaction where the reaction rate is dependent on the concentrations of water [H_2O] and ester bonds [E] catalyzed by acid, including organic acids [COOH]. Hydrolysis of PLA is known to be an autocatalytic reaction, where generated acid groups catalyze further hydrolysis, according to Scheme S.1.

$$[R1-E-R2]+[H_2O]+[COOH]\rightarrow[R1-COOH]+[OH-R2]+[COOH] \qquad \text{(SI.1)}$$

Here R1 and R2 represent the polymer chain on either side of the ester bond, E.

The rate of the above autocatalytic reaction can be described by Equation SI.2.

$$-r_{[E]}=-\frac{dC_E}{dt}=kC_EC_{H_2O}C_{COOH} \qquad \text{(SI.2)}$$

Here $r_{[E]}$ is the reaction rate, k is the ester hydrolysis rate constant, C_E is the concentration of ester bonds, C_{H_2O} and C_{COOH} represents the concentration of water and acid, respectively. In the present treatment, all ester bonds are treated the same and assumed to have identical reactivities.

The reaction stoichiometry allows the concentrations of all species to be expressed in terms of the initial concentration of ester bonds, $C_E(t=0)$, and their fractional conversion, $X=C_E(t)/C_E$ $(t=0)=C_E(t)/C_E(0)$. Simple substitution gives, $C_E(t)-C_E(0)=C_E(0)(X-1)$ which is rearranged and simplified to $C_E(t)=C_E(0)+C_E(0)(X-1)=C_E(0)X$. For each ester bond broken, a water molecule is consumed, therefore the moles of water consumed can be expressed in concentration by $C_{H_2O}(t)-C_{H_2O}(0)=C_E(t)-C_E(0)=C_E(0)(X-1)$. Rearrangement gives

$C_{H_2O}(t) = C_{H_2O}(0) + C_E(0)(X-1)$. Introducing the ratio of concentrations at time t=0 ($a = C_{H_2O}(0)/C_E(0)$) allows the water concentration to be expressed in terms of the ester bond conversion, $C_{H_2O}(t) = aC_E(0) + C_E(0)(X-1) = C_E(0)(a+X-1)$. The same line of argument and the corresponding definition, $b = C_{COOH}(0)/C_E(0)$gives $C_{COOH}(t) - C_{COOH}(0) = -(C_E(t) - C_E(0)) = -C_E(0)(X-1) = C_E(0)(1-X)$, $C_{COOH}(t) = C_{COOH}(0) + C_E(0)(1-X) = bC_E(0) + C_E(0)(1-X) = C_E(0)(b+1-X)$. Substitution for all concentrations in terms of the ester conversion into Eqn. SI.2 provides the result is SI.3 which may be simplified to SI.4.

$$-r_{[E]} = -\frac{d}{dt}\{C_E(0)X\} = k\, C_E(0)X\, C_E(0)(a+X-1)C_E(0)(b+1-X) \qquad \text{SI.3}$$

$$-\frac{dX}{dt} = k\, {C_E}^2(0)X(a+X-1)(b+1-X) \qquad \text{SI.4}$$

This differential equation may be separated, expanded in partial fractions, and then integrated. The result is Eqn. SI.5

$$\frac{\ln(X)}{(1-a)(b+1)} - \frac{[\ln(a-1+X) - \ln(a)]}{(1-a)(b+a)} + \frac{[\ln(b+1-X) - \ln(b)]}{(a+b)(b+1)} = k\, {C_E}^2(0)\, t = k't \qquad \text{SI.5}$$

Where $k' = k\, {C_E}^2(0)$, is again the apparent rate constant. The left hand side of Equation SI.5 is represented as Γ in Figure 4b of the main manuscript.

Conversion of PLA hydrolysis at the initial stage. The conversion of ester bonds by chain scission can be expressed as the molecular weight change of PLA at the initial hydrolysis stage, which corresponds to increased numbers of chains. The initial concentration is defined as,

$$C_E(0) = (\frac{m_0}{M_R} - \frac{m_0}{M_0})\frac{1}{V} \qquad \text{SI.6}$$

Where m_0 is the initial mass of PLA fibers, M_R is the molecular weight of repeat unit which is equal to 72 g/mol, V is the total volume. One cleaved ester bond generates one *new* PLA molecule with lower molecular weight. At a specific time, the consumed amount of ester bonds is expressed as,

$$C_{\mathrm{E,consumed}}(t) = \left(\frac{m_t}{M_t} - \frac{m_o}{M_0}\right)\frac{1}{V} \qquad \text{SI.7}$$

Where $\boldsymbol{m_t}$ and $\boldsymbol{M_t}$ is the mass and molecular weight of PLA at time $\boldsymbol{t}$, $\boldsymbol{C_{\mathrm{E,consumed}}(t)}$ is the consumed amount of ester bonds. At the initial hydrolysis stage, the mass loss is negligible, so $\boldsymbol{m_t} \approx \boldsymbol{m_0}$. The fractional conversion of ester bonds at time $\boldsymbol{t}$ is expressed as,

$$X = \frac{C_E(t)}{C_E(0)} = 1 - \frac{C_{\mathrm{E,consumed}}(t)}{C_E(0)} = 1 - \frac{M_R}{M_0 - M_R}\left(\frac{M_0}{M_t} - 1\right) \qquad \text{SI.8}$$

Upon substitution into Equation SI.5, the result is SI.9.

$$\frac{\ln\left[1-\frac{M_R}{M_0-M_R}\left(\frac{M_0}{M_t}-1\right)\right]}{(1-a)(b+1)} - \frac{\ln\left[a-\frac{M_R}{M_0-M_R}\left(\frac{M_0}{M_t}-1\right)\right]-\ln(a)}{(1-a)(b+a)} + \frac{\ln\left[b+\frac{M_R}{M_0-M_R}\left(\frac{M_0}{M_t}-1\right)\right]-\ln(b)}{(a+b)(b+1)} = k\, C_E{}^2(0)\, t \qquad \text{SI.9}$$

Using equation SI.9 to analyze the measured molecular weight of PLA versus time, gives hydrolysis rate constants; Figure SI.1 presents the data fits.

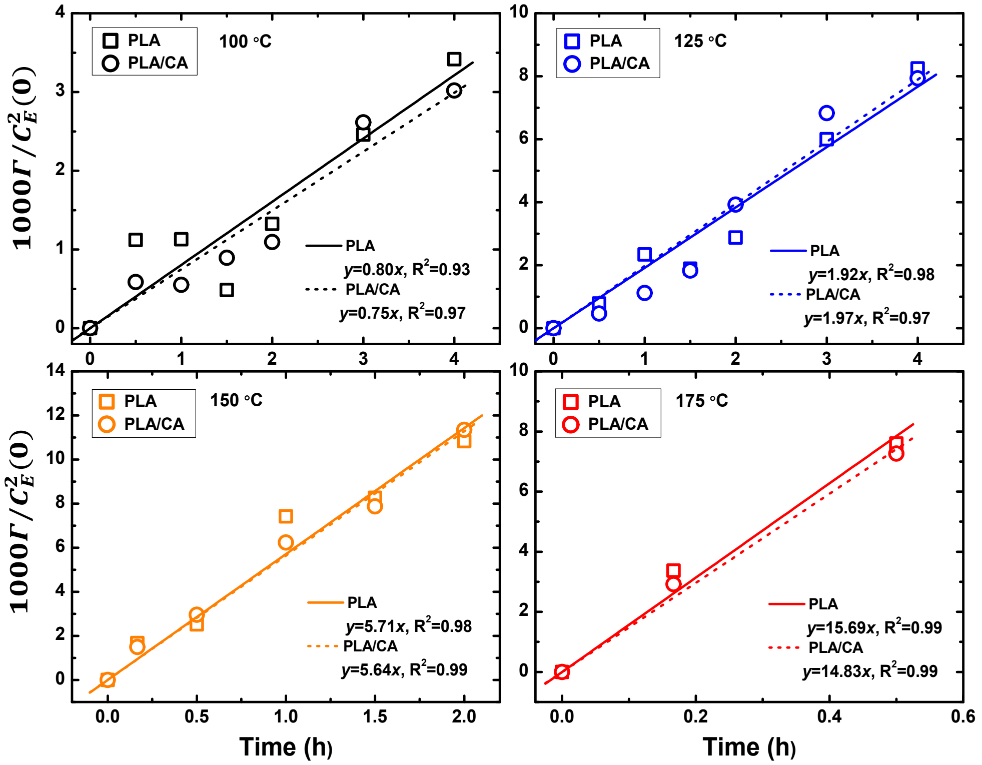

Figure SI.1. Rate constants from PLA molecular weight data; Γ represents the left hand side of equation SI.9; a) 100ºC, b) 125ºC, c) 150ºC , and d) 175 ºC.

Reported apparent hydrolysis rate constant of PLA based on molecular weight changes from literature are plotted in Figure SI.2a.[1-20] The dependence of the apparent hydrolysis rate constant on temperature from the literature in the temperature range of 37 to 250 ºC can be described by the Arrhenius relationship; this result is presented in Figure SI.2b. The obtained activation energy for PLA hydrolysis is 16.7 kcal/mol in the temperature range of 37 to 250 ºC, which agrees with the value of E_a = *14 +/- 1.4 kcal/mol* found in the present study.

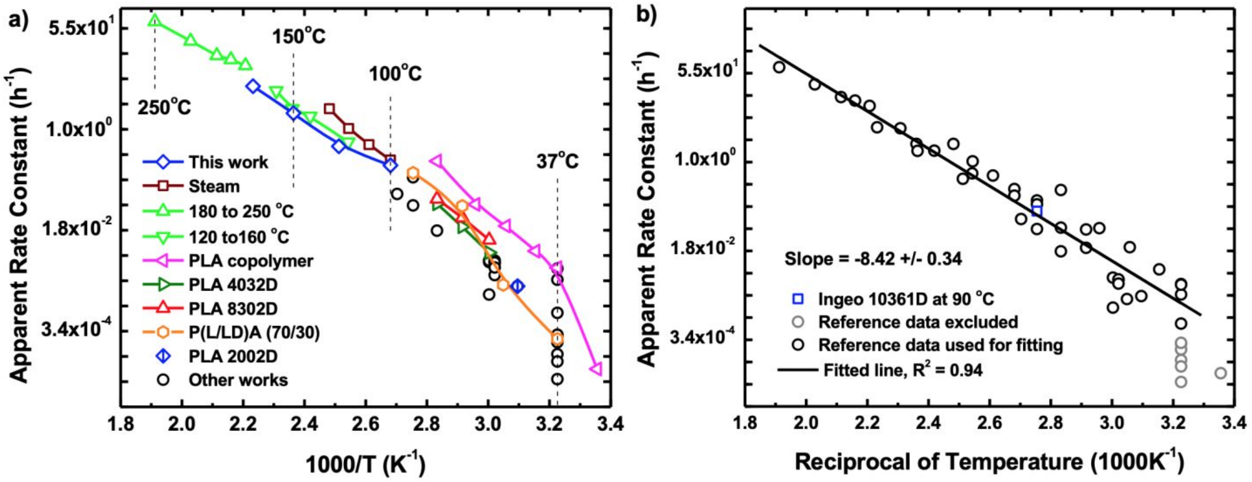

Figure SI.2. a) Apparent hydrolysis rate constant of PLA based on molecular weight changes reported in the literature, [1-20] and b) linear regression analysis of reported data excluding the indicated data points.

Hydrolysis of PLA 10361D (Ingeo™) microplastics. Further verification and applicability of the results is possible by hydrolyzing a commercial grade PLA (Ingeo™ 10361D). Micron sized plastic particles were fabricated by a hammer mill giving an average particle size of 490 (± 160) µm (Figure S1a). Hydrolysis of PLA 10361D microplastics were conducted as the same procedure as PLA and CA fibers at 90 ºC for varying times. The plots of $M_n(t)/M_n(0)$ versus time are presented in Figure S1b. at 90 ºC. Using only molecular weight measurements, the apparent hydrolysis rate constant for PLA 10361D is 0.11 ($\pm$0.01) h^{-1} and the calculated apparent rate constant is 0.09 h^{-1} .

This value is shown in Figure S1b and falls on the same line as the smaller particles demonstrating that the size regime for reaction rate based conversion is reached.

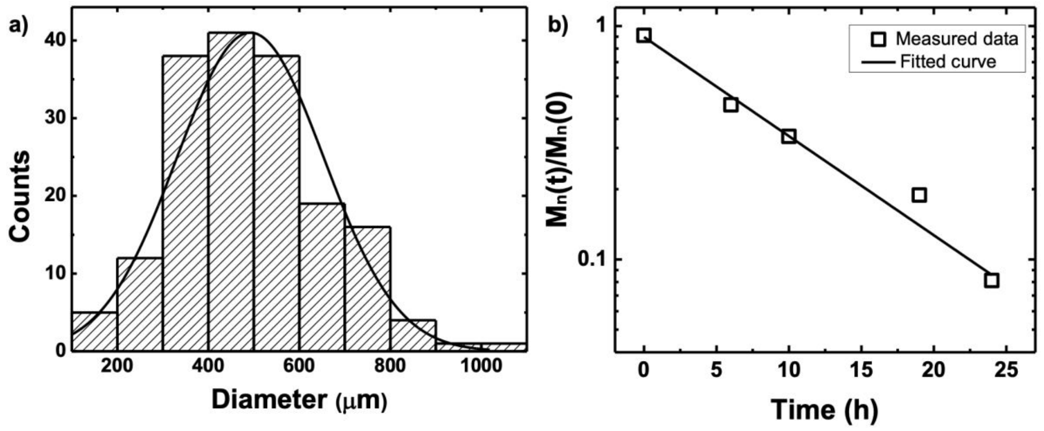

Figure SI.3. Hydrolysis of commercial grade PLA. a) Particle size distribution and b) exponential behavior of $M_n(t)/M_n(t)$ versus time.

References:

1. Tsuji, H.; Ikarashi, K. In vitro hydrolysis of poly(L-lactide) crystalline residues as extended-chain crystallites. Part I: long-term hydrolysis in phosphate-buffered solution at 37 degrees C. *Biomaterials* **2004,** 25, (24), 5449-5455.
2. Tsuji, H.; Nakahara, K.; Ikarashi, K. Poly(L-Lactide), 8. High-temperature hydrolysis of poly(L-Lactide) films with different crystallinities and crystalline thicknesses in phosphate-buffered solution. *Macromolecular Materials and Engineering* **2001,** 286, (7), 398-406.
3. Cha, Y.; Pitt, C. The biodegradability of polyester blends. *Biomaterials* **1990,** 11, (2), 108-112.
4. Tsuji, H.; Ikada, Y. Blends of aliphatic polyesters. II. hydrolysis of solution-cast blends from poly(L-lactide) and poly(1-caprolactone) in phosphate-buffered solution. *Journal of Applied Polymer Science* **1998,** 67, (3), 405-415.
5. Tsuji, H.; Mizuno, A.; Ikada, Y. Properties and morphology of poly(L-lactide). III. Effects of initial crystallinity on long-term in vitro hydrolysis of high molecular weight poly(L-lactide) film in phosphate-buffered solution. *Journal of Applied Polymer Science* **2000,** 77, (7), 1452-1464.
6. Mohd-Adnan, A.-F.; Nishida, H.; Shirai, Y. Evaluation of kinetics parameters for poly(l-lactic acid) hydrolysis under high-pressure steam. *Polymer Degradation and Stability* **2008,** 93, (6), 1053-1058.
7. Huang, Y.; Zhang, C.; Pan, Y.; Zhou, Y.; Jiang, L.; Dan, Y. Effect of NR on the hydrolytic degradation of PLA. *Polymer Degradation and Stability* **2013,** 98, (5), 943-950.

8. Tsuji, H.; Ikarashi, K. In vitro hydrolysis of poly(l-lactide) crystalline residues as extended-chain crystallites: II. effects of hydrolysis temperature. *Biomacromolecules* **2004,** 5, (3), 1021-1028.
9. Tsuji, H.; Daimon, H.; Fujie, K. A new strategy for recycling and preparation of poly(L-lactic acid): hydrolysis in the melt. *Biomacromolecules* **2003,** 4, (3), 835-840.
10. Tsuji, H.; Saeki, T.; Tsukegi, T.; Daimon, H.; Fujie, K. Comparative study on hydrolytic degradation and monomer recovery of poly(l-lactic acid) in the solid and in the melt. *Polymer Degradation and Stability* **2008,** 93, (10), 1956-1963.
11. Yuan, X.; Mak, A. F. T.; Yao, K. Comparative observation of accelerated degradation of poly(l-lactic acid) fibres in phosphate buffered saline and a dilute alkaline solution. *Polymer Degradation and Stability* **2002,** 75, (1), 45-53.
12. Mainil - Varlet, P.; Curtis, R.; Gogolewski, S. Effect of in vivo and in vitro degradation on molecular and mechanical properties of various low-molecular-weight polylactides. *Journal of Biomedical Materials Research* **1997,** 36, (3), 360-380.
13. Zhang, X.; Espiritu, M.; Bilyk, A.; Kurniawan, L. Morphological behaviour of poly(lactic acid) during hydrolytic degradation. *Polymer Degradation and Stability* **2008,** 93, (10), 1964-1970.
14. Gorrasi, G.; Pantani, R. Effect of PLA grades and morphologies on hydrolytic degradation at composting temperature: Assessment of structural modification and kinetic parameters. *Polymer Degradation and Stability* **2013,** 98, (5), 1006-1014.
15. Bergsma, J. E.; Rozema, F. R.; Bos, R. R. M.; Boering, G.; Joziasse, C. A. P.; Pennings, A. J. In vitro predegradation at elevated temperatures of poly(lactide). *Journal of Materials Science: Materials in Medicine* **1995,** 6, (11), 642-646.
16. Xu, H.; Yang, X.; Xie, L.; Hakkarainen, M. Conformational footprint in hydrolysis-induced nanofibrillation and crystallization of poly(lactic acid). *Biomacromolecules* **2016,** 17, (3), 985-995.
17. Rodriguez, E. J.; Marcos, B.; Huneault, M. A. Hydrolysis of polylactide in aqueous media. *Journal of Applied Polymer Science* **2016,** 133, (44), 44152.
18. Mitchell, M. K.; Hirt, D. E. Degradation of PLA fibers at elevated temperature and humidity. *Polymer Engineering & Science* **2015,** 55, (7), 1652-1660.
19. Agrawal, C. M.; Huang, D.; Schmitz, J. P.; Athanasiou, K. A. Elevated temperature degradation of a 50:50 copolymer of PLA-PGA. *Tissue Engineering* **1997,** 3, (4), 345-352.
20. Lyu, S.; Schley, J.; Loy, B.; Lind, D.; Hobot, C.; Sparer, R.; Untereker, D. Kinetics and time-temperature equivalence of polymer degradation. *Biomacromolecules* **2007,** 8, (7), 2301-2310.
